# How Has the Free Obstetric Care Policy Impacted Unmet Obstetric Need in a Rural Health District in Guinea?

**DOI:** 10.1371/journal.pone.0129162

**Published:** 2015-06-05

**Authors:** Alexandre Delamou, Dominique Dubourg, Abdoul Habib Beavogui, Thérèse Delvaux, Jacques Seraphin Kolié, Thierno Hamidou Barry, Bienvenu Salim Camara, Mary Edginton, Sven Hinderaker, Vincent De Brouwere

**Affiliations:** 1 Centre national de formation et de recherche en santé rurale de Maferinyah, Forécariah, Guinea; 2 Ecole de Santé Publique, Université Libre de Bruxelles, Bruxelles, Belgium; 3 Observatoire Wallon de la Santé, Namur, Belgium; 4 Hopital Prefectoral de Kissidougou, Kissidougou, Guinea; 5 The International Union Against Tuberculosis and Lung Disease, Paris, France; 6 School of Public Health, University of the Witwatersrand, Johannesburg, South Africa; 7 University of Bergen, Bergen, Norway; 8 Women and Child Health Research Center, Institute of Tropical Medicine, Antwerp, Belgium; Johns Hopkins University, UNITED STATES

## Abstract

**Introduction:**

In 2010, the Ministry of Health (MoH) of Guinea introduced a free emergency obstetric care policy in all the public health facilities of the country. This included antenatal checks, normal delivery and Caesarean section.

**Objective:**

This study aims at assessing the changes in coverage of obstetric care according to the Unmet Obstetric Need concept before (2008) and after (2012) the implementation of the free emergency obstetric care policy in a rural health district in Guinea.

**Methods:**

We carried out a descriptive cross-sectional study involving the retrospective review of routine programme data during the period April to June 2014.

**Results:**

No statistical difference was observed in women’s sociodemographic characteristics and indications (absolute maternal indications versus non-absolute maternal indications) before and after the implementation of the policy. Compared to referrals from health centers of patients, direct admissions at hospital significantly increased from 49% to 66% between 2008 and 2012 (p = 0.001). In rural areas, this increase concerned all maternal complications regardless of their severity, while in urban areas it mainly affected very severe complications. Compared to 2008, there were significantly more Major Obstetric Interventions for Maternal Absolute Indications in 2012 (p<0.001). Maternal deaths decreased between 2008 and 2012 from 1.5% to 1.1% while neonatal death increased from 12% in 2008 to 15% in 2012.

**Conclusion:**

The implementation of the free obstetric care policy led to a significant decrease in unmet obstetric need between 2008 and 2012 in the health district of Kissidougou. However, more research is needed to allow comparisons with other health districts in the country and to analyse the trends.

## Introduction

The maternal mortality rate in the world has decreased by almost half between 1990 and 2013 but, the majority of low-income countries, especially in West and Central Africa will not achieve the Millennium Development Goals 4 and 5 in 2015 [1]. According to the World Health Organization (WHO) recent estimates, the Sub-Saharan Africa region alone accounts for 62% (179 000) of the global maternal deaths, followed by Southern Asia (69 000) [2].

There is evidence that availability of Major Obstetric Interventions along with reduced transport time and reduced financial barriers to care has the potential to reduce maternal mortality rates [3].

To address this situation, many countries in Africa have implemented user fees exemption policies for emergency obstetric care to reduce financial barriers to access health services [4]. These initiatives aim at improving maternal and child health through equitable access to health services, especially for the most vulnerable groups [5–7]. In fact most of the time women have to pay for care at delivery and when any pregnancy related complication occurs and to some extent during antenatal visits. This is often the case when the woman has to undergo a caesarean section, leading to out of pocket payments that expose households to poverty, especially in rural settings [8]. Even though barriers other than financial may exist to influence the high maternal mortality rate in in many low income countries, user fee exemption policies were seen as a mean to facilitate access to maternal care [4].

To assess the impact of these policies in improving the coverage of emergency obstetric care, the concept of Unmet Obstetric Needs (UON) was developed in Morocco [9] and used in different contexts [10–13].

The UON concept is a tool and an appropriate approach to evaluate inequalities in accessing lifesaving obstetric surgery [14]. It also helps in advocacy towards policy makers and health personnel for concerted action aimed at improving the response of health services to health care demand, especially when it relates to emergency obstetric care. For example, a study of UON in rural Mali showed that deficits for the whole district were 110 missing Major Obstetric Interventions, which was interpreted as the number of women who should have benefited from a lifesaving intervention but did not, meaning that they probably died or suffered very severe complication [10]. The findings helped raise awareness of the district management team for a better provision of maternal health. In addition, an increased rate of UON indicator, namely Major Obstetric Interventions for Absolute Maternal Indications, has been reported after the introduction of fees exemption policy in Burkina Faso [15]. However, studies in Tanzania have reported wide rural/urban disparities in access to emergency obstetric care and as a result in UON indicators [16, 17].

In 2010, the Ministry of Health (MoH) of Guinea introduced a free emergency obstetric care policy in all the public health facilities of the country. This included antenatal care, normal delivery and Caesarean section. However, since its implementation, many questions remain as to whether the policy has benefited to poor and rural women or whether the implementation of this strategy has contributed to decrease the maternal mortality thanks to an increased access to life saving obstetric interventions. The Guinean 2012 Demographic and Health Survey reported a still high Maternal Mortality Ratio of 724 per 100 000 live births [18].

While the concept of UON has been implemented in many countries and the maternal care fee exemption policies documented, little is known about how user fee exemption has impacted UON indicators in Guinea.

Using data of women who underwent obstetric interventions at the district hospital of Kissidougou in 2008 (before the implementation of the policy) and in 2012 (after the implementation) we report on: 1) the demographic and clinical characteristics of patients before and after the adoption of the policy, 2) the numbers and percentage of women who needed a Major Obstetric Intervention but did not have it (known as unmet obstetric needs) before and after the adoption of the policy by residence (urban vs rural), 3) the maternal and child health outcomes before and after the adoption of the policy.

## Methods

### The UON concept

**Fig 1:**
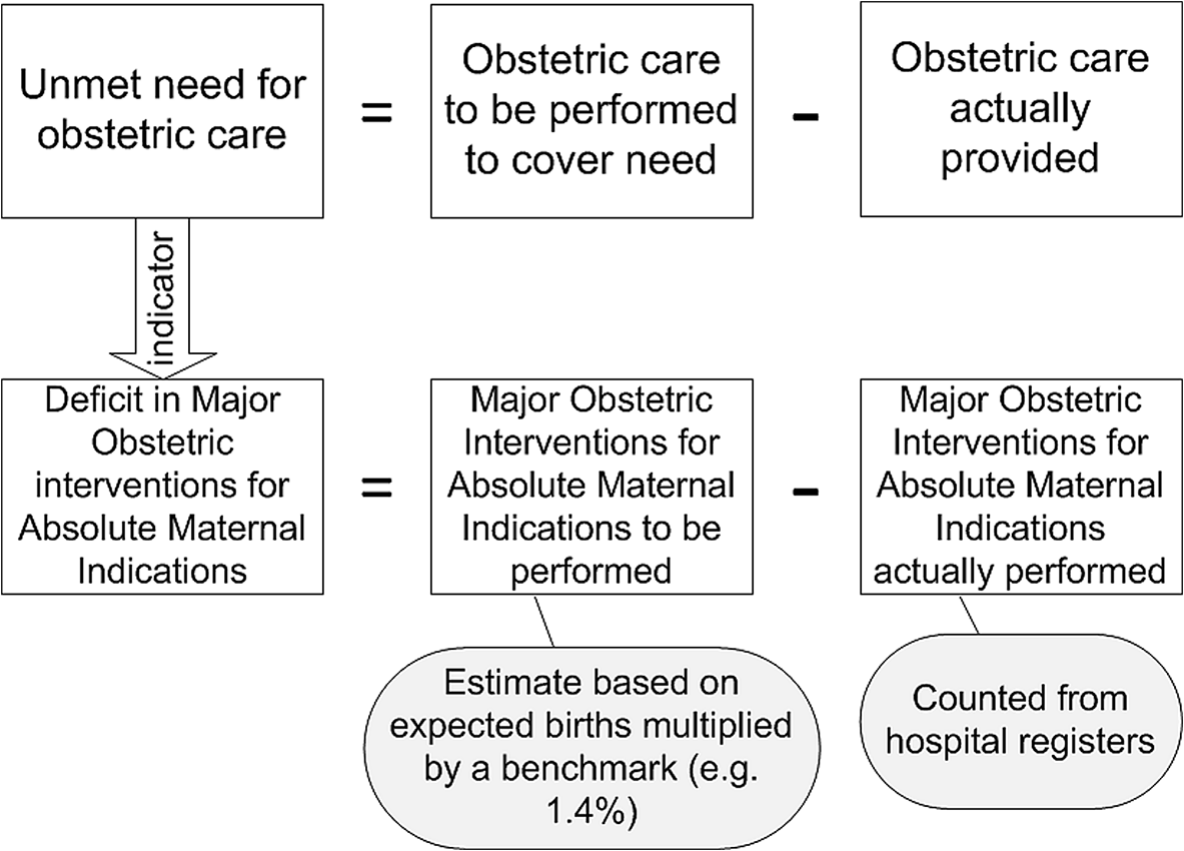
The Concept of Unmet Obstetric Needs (UON). (adapted from UON Tackling Unmet Need for Major Obstetric Interventions. Concepts, General Principles and International Network. ITGPress, 1999. http://www.uonn.org/pdf/Guide1.pdf)

The approach and strategy of UON has already been described [9,19]. The UON indicator gives an estimate of the gap between the expected Major Obstetric Interventions (MOI) required for Absolute Maternal Indications (AMI) in given well-defined population, such as a health district (needs), and the actual delivery of these services to this population (Fig 1).

Studies using the UON approach in different contexts have taken a thresholds ranging from 1% to 2% [17, 20, 21] of total deliveries as the minimum expected number of MOI for AMI. As no previous evaluation of the UON has been conducted in Guinea, we have decided, based on the literature and experts’ advice to use the median threshold, suggested by the UON Network, of 1.4% of all deliveries for this study [14].

### Study design

This was a descriptive cross-sectional study involving the retrospective review of routine programme data.

### Setting

#### General setting

With an estimated 12 million inhabitants in 2012, Guinea is a low income country where 65% of the population lives in rural areas and 55% of the population earn less than $300 per year [18, 22]. The maternal mortality ratio has decreased from 980 to 724 per 100 000 Live Births between 2005 and 2012 but still remain high while the use of contraception is low (only 7% of women of reproductive age were using modern contraceptives in 2012) [18, 23]. In 2012, the estimated number of deliveries was 435,000 and the proportion of deliveries with skilled attendant was 45.3% at national level with a wide urban/rural disparity (83.9% versus 31.6% respectively) [18].

#### Study setting

The health district of Kissidougou covers a population of about 290,000 inhabitants [24]. The district hospital serves as the reference hospital for 17 public health centres. This hospital is the only one that provides obstetric interventions including caesarean section in the district. The free obstetric care policy consisted of free access to antenatal care, normal delivery and c-section for all women in all the public health facilities. Before this policy, hospital expenses were paid in cash by women and their families. With the free obstetric care policy launched in 2010, the MoH provided funding and equipment to the hospital and Health Centers. International medical organizations such as EngenderHealth provided additional technical and financial support to upgrade infrastructures and train the medical staff of the maternity unit as part of the Fistula Care Project [25]. In 2011, the MoH offered an emergency transportation to the district hospital as part of the policy. The hospital comprises 48 beds in three inpatient departments (paediatrics, surgery and medicine), a 27-bed maternity unit with an additional 14-bed fistula unit), an emergency service, a laboratory, dentistry and a service of Medical Imaging (x-ray). The hospital has two obstetricians, two surgeons, two midwives, two nurses and seven auxiliary health providers that run a twenty-four hour care in the delivery ward.

### Study population and participants

All women who underwent obstetric intervention at the district hospital of Kissidougou between 1st January 2008 to 31 December 2008 (before) and 1st January 2012 to 31 December 2012 (after) were included in the study.

### Data variables, sources of data and data collection

Data were collected between April and June 2014. The socio-demographic and clinical characteristics of patients included age, parity, residence (rural/urban), mode of admission (direct admission/referral from health centres), and maternal indication for C-section or MOI. Maternal and neonatal health outcomes were woman alive (yes/no), child alive (yes/no), as reported in hospital medical records. C-section rate was calculated by using the number of c-section performed during the year (numerator) and the expected number of births in the same year (denominator).Unmet obstetric needs (needs of MOI–actual MOI) were computed as follows: MOI represented the number of surgical procedures performed (Fig 1) and Absolute Maternal Indication represented the diagnoses established by the health care team (Fig 1). The expected MOI for AMI was estimated using the expected number of births in the district for each year and the UON threshold of 1.4% [14]. We derived the number of MOI for AMI actually performed per year from our dataset and, we calculated the unmet need (Fig 1). Structured forms were used to extract the study-related data from patient hospital records kept at the maternity.

### Data analysis

Data collected from patient’s files was double entered by two independent encoders into a file using EpiData Entry software (version 3.1, EpiData Association, Odense, Denmark). The two data files were compared and discordances resolved by cross-checking with the paper registers. Data were analysed using STATA 13 software (STATA Corporation, College Station, TX, USA). Frequencies (%) were calculated to describe patients’ characteristics and maternal and child outcomes.

Pearson’s Chi Square (χ2) and Fischer’s exact test were used to compare proportions of study outcomes between the two years. The level of significance was set at p = 0.05 with a 95% confidence intervals

### Ethics approvals

Ethics approval was obtained from the Guinean National Ethics Committee for Health Research and the study satisfied the ethics criteria of the MSF Ethics Review Board (Geneva, Switzerland) and the Ethics Advisory Group of the International Union Against Tuberculosis and Lung Disease, Paris, France, for studies using routinely collected programme data. This being a retrospective study in hospital setting, consent was not obtained. However, patient records/information was anonymized and de-identified prior to analysis.

## Results

### Sociodemographic and clinical characteristics

In 2008 and 2012, 206 and 361 women respectively underwent a Major Obstetric Interventions at the referral district hospital of Kissidougou. The demographic and clinical characteristics of patients before and after the adoption and implementation of the free obstetric care are shown in Table 1.

**Table 1:** Demographics and clinical characteristics of patients before and after the adoption of the policy at Kissidougou District Hospital in 2008 and 2012.

Variable	2008	2012	P-value*
	N(%)	N(%)	
**Total**	**206**	**361**	
**Age [years]**			0.24
15–24	94 (45.6)	173 (47.9)	
25–34	87 (42.2)	130 (36.0)	
35–44	25 (12.1)	58 (16.1)	
Median [IQR]	25 [20–30]	25 [19–30]	
**Pregnancies**			0.38
1–5	169 (82.0)	291 (80.6)	
≥ 6	37 (18.0)	70 (19.4%)	
Mean ± SD	3.29 ± 2.34	3.36 ± 2.38	
**Parity**			0.81
0 birth	61 (29.6)	98 (27.1)	
1–5 births	119 (57.8)	218 (60.4)	
≥ 6 birth	26 (12.6)	45 (12.5)	
Mean ± SD	2.35 ± 2.37	02.36 ± 2.36	
**Residence**			
Rural	143 (69.4)	237 (65.6)	0.36
Urban	63 (30.6)	124 (34.4)	
**Mode of admission**			0.001
Direct	102 (49.5)	231 (64.0)	
Referred	104 (50.5)	130 (36.0)	
**Indication**			0.191
AMI	107 (51.9)	208 (57.6)	
Non-AMI	99 (48.1)	153 (42.4)	
***Absolute Maternal Indication*** **			
Cephalo Pelvic Disproportion	76 (71.0)	146 (70.2)	0.100
Uterine rupture and pre-rupture	22 (20.6)	27 (13.0)	
Placenta praevia and abrutio placenta	9 (8.4)	26 (12.5)	
Malpresentation	1 (0.9)	9 (4.3)	
***Non-Absolute Maternal Indication*** ***			0.151
Previous c-section	35 (35.4)	56 (36.6)	
Dynamic dystocia	30 (30.3)	26 (17.0)	
Fœtal indication	19 (19.2)	42 (27.5)	
Hypertensive disorder	0 (0)	1 (0.7)	
Vesico-vaginal fistula	2 (2.0)	5 (3.3)	
Other	12 (12.1)	23 (15.0)	
**Post-op complications**			
***Absolute Maternal Indication***			0.589
Yes	25 (23.4)	37 (17.8)	
No	82 (76.6)	171 (82.2)	
***Non-Absolute Maternal Indication***			0.008
Yes	8 (8.1)	2 (1.3)	
No	91 (91.9)	151 (98.7)	

* Chi square test for two independent proportions.

** Percentages are expressed with AMI as denominator.

***Percentages are expressed with Non-AMI as denominator.

No statistical difference was observed in women’s age, number of previous pregnancies, parity, residence (rural versus urban) and indication (Absolute Maternal Indication versus non-absolute maternal indication) before and after the implementation of the policy. In both years, the median age of women was 25 years (interquartile range (IQR) 20–30) with almost one out of two aged 15–24 years. Two out of three women lived in rural area and the majority of women were multiparous.

Compared to referrals from health centers of patients, direct admissions at hospital significantly increased from 49% to 64% between 2008 and 2012 (p = 0.001). In rural areas, this increase concerns all maternal complications regardless of their severity, while in urban areas it mainly affects very severe complications.

Post-operative complications significantly dropped between 2008 and 2012 among patients presenting with non-Absolute Maternal Indication (p = 0.008). No significant change was observed in patients with Absolute Maternal Indication. The most frequent Absolute Maternal Indications before and after the policy were cephalo-pelvic disproportion (71% versus 70%), followed by Uterine Rupture and Pre-Rupture. Previous C-section was the main non-Absolute Maternal Indication in 2008 (35%) and 2012 (37%).

### Major obstetric Intervention and Unmet Obstetric needs

**Fig 2:**
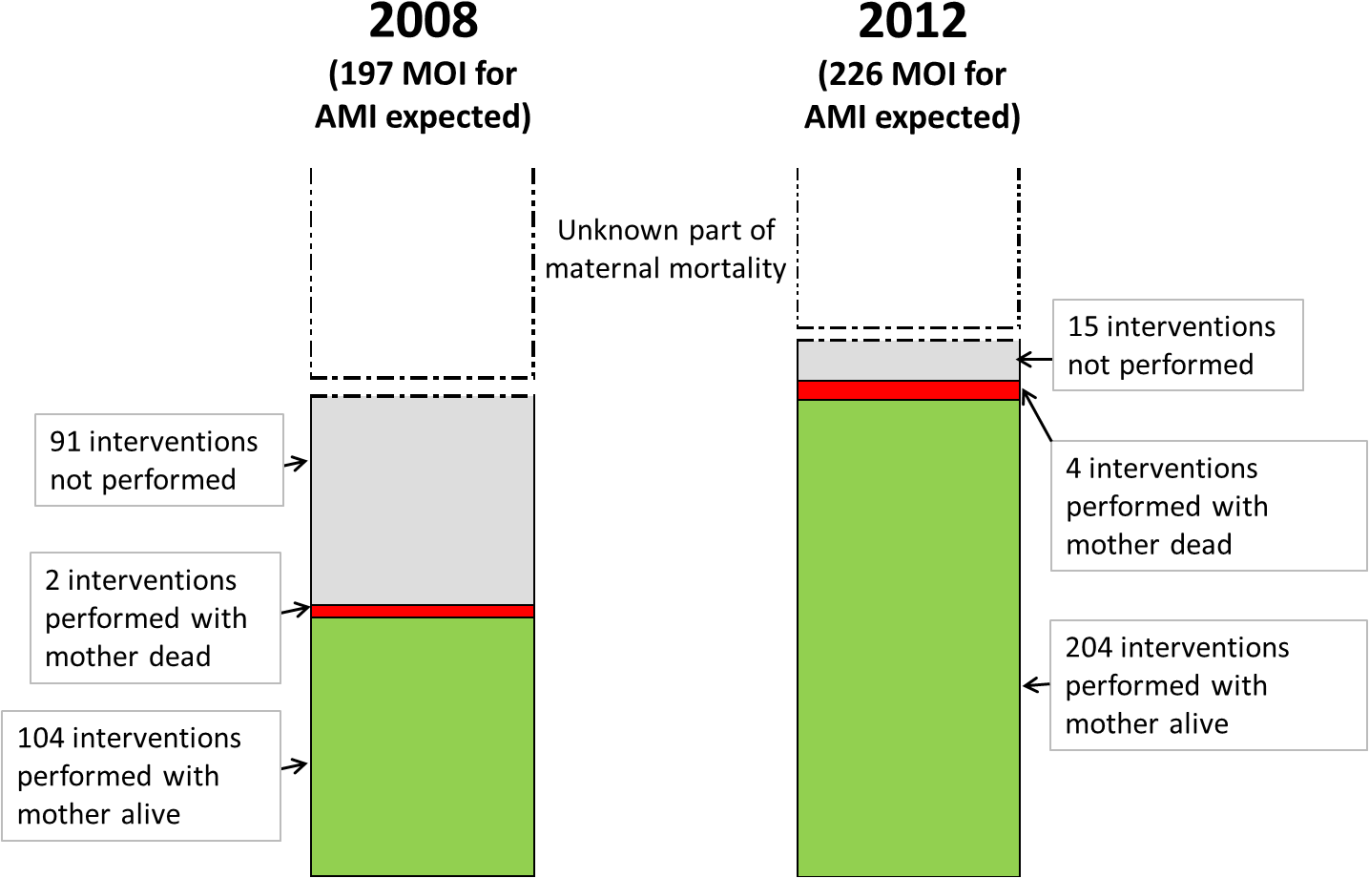
Numbers of women who needed, received (met Obstetric needs) and did not receive (unmet Obstetric needs) MOI for AMI before (2008) and after (2012) the adoption of the policy in the health district of Kissidougou, Guinea.

**Fig 2** presents the numbers and percentage of women who received a Major Obstetric Intervention for Maternal Absolute Indication before and after the adoption of the policy. Compared to 2008, there were significantly more MOI for AMI in 2012 (p<0.001, Fig 2.). Of the 226 MOI for AMI expected in 2012, 208 were performed (93%, 95%CI 89%-96%) while only 106 out of 197 expected (54%, 95% CI 44%-63%) were carried out in 2008.

In numbers, 91 Major Obstetric Interventions that should be performed to save the life of a woman were not performed in 2008 compare to 15 in 2012, Fig 2.

This reduction of missing MOI for AMI was statistically significant for both women coming from rural and urban areas. This reduction of missing MOI for AMI was statistically significant for both women coming from rural and urban areas. The reduction was higher in rural setting where the coverage of MOI for AMI increased from 76 (55%) to 154 (99%) (p<0.001) compare to urban settings where it raised from 31 (53%) to 54 (81%), (p = 0.006)

### C-section rate and Maternal and Child outcomes

**Table 2** shows the maternal and neonatal outcomes before and after the implementation of the fees exemption policy. Maternal death after MOI decreased between 2008 and 2012 from 1.5% to 1.1% while, neonatal death increased from 12% in 2008 to 15% in 2012. These changes in maternal and neonatal outcomes were not statistically significant. No significant change was observed in C-section rate as a result of the free exemption policy even though the overall rate increased (1.5% to 2.3%) as it did in both rural (1.5% to 2.1%) and urban area (1.5% to 2.7%).

Finally, the MOI per 100 births increased from 0.8% in 2008 to 1.3% in 2012 (p<0.001).

**Table 2:** Maternal and child outcomes before and after the adoption of the policy at Kissidougou District Hospital in 2008 and 2012.

Variable	2008	2012	P-value*
	N(%)	N(%)	
**Maternal outcome**			0.71
Alive	203 (98.5)	357 (98.9)	
Dead	3 (1.5)	4 (1.1)	
Total	206 (100.0)	361 (100.0)	
**Neonatal outcome**			0.31
Alive	182 (88.4	308 (85.3)	
Dead	24 (11.6)	53 (14.7)	
Total	206 (100.0)	361 (100.0)	
**Caesarean rate**			0.36
Rural	143 (1.5)	232 (2.1)	
Urban	63 (1.5)	129 (2.7)	
Total	206 (1.5)	361 (2.3)	
**MOI for AMI**			
Rural	76 (55.1)	154 (98.7)	< 0.001
Urban	31 (52.5)	54 (80.6)	0.006
Total	106 (53.8)	208 (93.3)	< 0.001

## Discussion

This is the first study from Guinea reporting on the impact of the free emergency obstetric care policy introduced in public health settings. The study showed a significant reduction of Unmet Obstetric Needs in the Kissidougou Health District between 2008 and 2012. The number of women referred to the hospital from health centers significantly decreased as a result of higher direct admissions. The higher proportion of direct admissions observed in our study can be explained by the large package of the free obstetric care policy which reduced financial barriers and allowed more women to benefit antenatal care, normal delivery and c-section for free in all public hospital. Direct admission in referral hospital can increase if women bypass primary care facilities for childbirth. In Tanzania for instance, Kruk et al [26] reported that four out of ten women who delivered in a health-care facility in rural parts country chose to deliver in a hospital or a health centre rather than at local primary care clinic even if they lived near a functioning clinic with delivery services.

Our results show an increase of met obstetric need for women in the whole health district; especially for those living in rural areas. This may be due to factors including the implementation of the policy but also to the provision of an ambulance to the hospital, sensitization campaign developed at community level by Engenderhealth or improvement of quality of care in the maternity ward through the Fistula Care Project [25]. The positive impact of fees exemptions policies have been reported from different settings in Africa [27,28]. In Burkina Faso where 80% of the direct costs of normal deliveries were subsidized between 2006 and 2010, Ride et al [27] found a significant decrease in the prevalence of households with excessive medical expenses (from 4.0% to 0.9%) and concluded that all categories of the population benefited from this policy, including the poorest even if only 10% of women from the poorest quintile were exempted. In contrary, in the context of full elimination of fees for facility-based births in another health district in Burkina Faso, Ben Ameur et al [29] reported more benefits for the poorest.

Boudreaux et al [30] using data from before and after a pilot implementation of user fees elimination found that, in Laos, women living in the pilot areas were more likely to seek professional care for delivery than those in control areas after two years of follow-up while these patterns were reverse before the pilot.

The increase of the MOI for AMI per 100 expected births observed in 2012 (0.8% to 1.5%) suggests that once financial barriers to emergency obstetric care are removed, women with severe complications are less hesitant to seek care at health facilities. Our findings were lower than that reported by Hunger et al [31] in Tanzania (rate of MOI for AMI per 100 expected births at 1.8%). However, they found a wide rural–urban disparities (1.4% vs. 3.3%) while in our study, the significant increase occurred in both rural areas (p<0.001) and urban areas (p = 0.05).

The relative increase in neonatal death observed might be due to a decrease in the quality of neonatal care as a result of the work overload of health workers. It might also be because the providers do not have the training to save new-borns or they may not have the life-saving equipment or both. The increase might also be due to the fact that women come soon enough for the mother's life to be saved, but not fast enough for the child to be saved. Given that direct admissions rose from rural areas, it is likely that women spend "more time" because of the distance, to get to the hospital, and this might affect the survival of the child. According to Ronsmans, some women only seek skilled care when they are ill, and they may do so too late for a midwife or doctor to be able to save the lives of the mothers or neonates [32]. In addition, because of the increased direct admissions, women who bypass primary care facilities do not receive care before arriving at the hospital or when they come, they delay before receiving adequate care for them and their newborns, especially when providers know that the woman will not pay for the care received [32,33]. However, in a case study conducted in eight countries from three continents, Richard et al. reported no significant changes in foetal outcomes in most of the settings but rather noted a small reduction [34].

Our findings should be considered with caution because they might have been influenced by other contextual factors. First, between 2007 and 2013, EngenderHealth (an international reproductive health non-governmental organization) established four dedicated centres for fistula management in Guinea, including the Kissidougou District hospital through the Fistula Care Project [25]. Fistula services were integrated in the maternity ward. As a result, the personnel of the hospital was reinforced, trained, equipped and monitored on a regular basis. This might have improved and maintained the quality and availability of services leading to more trust from the population and thus an increased use of maternity services including delivery. Second, the Fistula Care Project established Safe Motherhood Village Committees in ten villages whose members were involved in bringing up community awareness, birth preparedness and identification and referrals of high risk women to health facilities. This might have contributed to the increase of direct admissions observed. In fact an evaluation of community-level fistula prevention interventions in Guinea [35] shows that women living in communities where care-seeking behaviours are promoted through community-level cadres were two times more likely to attend at least four ANC visits, to deliver in a health facility, and to seek care for perceived complications than women in other communities. In a rural district setting in Burkina Faso, service users reported that the quality of care, leadership of health workers and strengthening relationships of trust with communities were among the reasons of the immediate increase in coverage of facility-based deliveries in their area [36].

The current national context characterized by the Ebola Virus Disease epidemic that has weakened the whole health system and probably the government ability to sustain funding of the free obstetric care policy raise questions about the sustainability of our findings. In addition, the Fistula Care project ended in September 2013. More research is needed to better understand factors that enabled the achievement of the results observed in Kissidougou and evaluate the impact of the Ebola epidemic on the free obstetric care policy and the level of UON in Guinea.

Our study has some limitations: 1) the estimate of the UON provided here could have been underestimated as some women living in the health district might have travelled for care in the neighbouring health districts for care, 2) this is a retrospective study in which we could not account for important individual level characteristics such as the level of education or socioeconomic status because of the lack of such data in hospital registers, 3) there is no national data that can allow comparison between health districts to see the variations and learn from different contexts. However the study has the following strengths: 1) the district referral hospital was the only one performing Major Obstetric Interventions in the district’s coverage area. Two other hospitals are situated at 89 km and 130 km, and it is likely that very few women have been able to go to one of these hospitals in case of complication. Our data, thus provide a correct estimate of the Unmet Obstetric Needs, 2) the use of the UON approach has been proven valid in other settings, 3) our results are reported according to the STROBE (Strengthening the Reporting of Observational Studies in Epidemiology) guidelines [37] and the study follows ethics considerations for operational research [38].

## Conclusion

Our study shows a significant decrease in unmet obstetric needs between 2008 and 2012 in the health district of Kissidougou. This followed the implementation of the free obstetric care policy in public services in an enabling environment. In our context, a national representative study of this kind could allow comparisons between districts and analysis of trends. This could also provide better comprehension of shortcomings usually reported in the implementation of government funded policies.

We agree with Hunger et al [31] that the rate of MOIs for AMIs is a powerful indicator of inequalities in access to obstetric care, and that it should be used on a regular basis to assess and monitor the functioning of the referral system in dealing with maternal complications in low resource setting.
